# Association of neuroimaging markers of cerebral small vessel disease with short-term outcomes in patients with minor cerebrovascular events

**DOI:** 10.1186/s12883-021-02043-9

**Published:** 2021-01-13

**Authors:** Xuemei Chen, Lin Wang, Junying Jiang, Yuanyuan Gao, Rui Zhang, Xiaoyuan Zhao, Tingwen Shen, Qi Dai, Junrong Li

**Affiliations:** 1 Department of Neurology, The Affiliated Jiangning Hospital with Nanjing Medical University, Nanjing, 211100 Jiangsu China; 2 Department of General Practice, The Affiliated Jiangning Hospital of Nanjing Medical University, Nanjing, 211100 Jiangsu China; 3 Department of Radiology, The Affiliated Jiangning Hospital with Nanjing Medical University, Nanjing, 211100 Jiangsu China

**Keywords:** White matter hyperintensity, Cerebral small vessel disease, Burden, Minor cerebrovascular events, Outcome

## Abstract

**Background:**

Increasing evidences have showed that neuroimaging markers of SVD can predict the short-term outcome of acute ischemic stroke (AIS). It is unclear that whether neuroimaging markers of SVD are also associated with short-term outcomes of minor cerebrovascular events. In the present study, we investigate neuroimaging markers of SVD in order to explore their roles in prediction of short-term outcome in patients with minor cerebrovascular events.

**Methods:**

Consecutive first-ever stroke patients (*n* = 546) from the Affiliated Jiangning Hospital of Nanjing Medical University were enrolled. A total of 388 patients were enrolled according to minor cerebrovascular events definition (National Institutes of Health Stroke Scale Score ≤ 3) and exclusion criteria. MRI scans were performed within 7 days of stroke onset, and then neuroimaging markers of SVD including WMH, lacunes, cerebral microbleeds (CMB), and perivascular spaces (PVS), SVD burden scores were assessed. We completed baseline characteristics and evaluated the relationships of short-term outcomes to SVD neuroimaging markers and SVD scores. The 90-day modified Rankin Scale (mRS) was thought as primary outcome and was dichotomized as good functional outcome (mRS 0–1) and poor outcome (mRS 2–6). Secondary outcomes were stroke progression and stroke recurrence.

**Results:**

Higher age, National Institutes of Health Stroke Scale (NIHSS) upon admission, lipoprotein-associated phospholipase A2 (LP-PLA2) and lacunes, Fazekas score were correlated with poor functional outcome (*P* < 0.05), But after adjusting for confounding variables, among the neuroimaging markers of cerebral small vessel disease, only Fazekas score (OR, 1.343; 95% confidence interval, 1.020–1.770; *P* = 0.036) was found to be associated with poor outcome at 90 days. Higher Fazekas and SVD scores were not associated with stroke progression or stroke recurrence.

**Conclusion:**

WMH can predict the poor functional outcome of minor cerebrovascular events. Adding other neuroimaging markers of SVD and total SVD burden score, however, does not improve the prediction, which indicated WMH can as neuroimaging markers for guiding the treatment of minor cerebrovascular events.

## Background

Cerebral small vascular disease (SVD) is an imaging, pathological and clinical syndrome caused by intracranial small vessel disease [1]. WMH, presented as high signal lesions on fluid-attenuated inversion recovery (FLAIR) sequence, is a common disease in the elderly [2] and has been proposed as a radiographic marker of SVD. Epidemiological studies revealed that about 70% of people over 65 years present with different degrees of WMH after undergoing magnetic resonance imaging (MRI) [3, 4]. The clinical significance of WMH is based on its robust associations with an increased risk of first or recurrent ischemic stroke [5, 6], intracerebral hemorrhage [7, 8], dementia [9, 10], and mortality [11].

However, WMH is just thought to be one of the MRI markers of SVD. Other standard definitions of SVD markers include lacunes, lobar and deep cerebral microbleeds (CMB), and enlarged perivascular spaces (PVS) [12]. WMH and SVD burden may signify a diminished capacity of cerebral tissue to withstand ischemia. Various neuroimaging markers of SVD could be poor prognostic markers for ischemic stroke survivors. However, to date only a few studies have combined these SVD features to capture total SVD burden and explore the association of them with the risk and severity of stroke, such as the SVD burden independently predicts higher acute ischemic stroke (AIS) risk [13] and more serious stroke [14, 15]. Minor cerebrovascular events (National Institutes of Health Stroke Scale Score ≤ 3) as an important part of AIS tend to recur and progress. And few studies have investigated the association of the SVD burden with short-term outcome in patients with minor cerebrovascular events.

We, therefore, aimed to undertake the present study to determine whether WMH or total SVD burden could be useful as a MRI marker for short-term outcome of minor cerebrovascular events.

## Methods

### Study population

In the prospective study, we analyzed data of minor cerebrovascular events patients, aged 45 to 85 years, enrolled from March 1, 2018 to March 1, 2020 from the department of neurology, Affiliated Jiangning Hospital of Nanjing Medical University. AIS patients were defined based on their clinical presentation and/or the presence of hyperintense lesion on Diffusion Weighted Imaging (DWI). For stroke patients with no DWI lesion, stroke diagnosis was confirmed by two experienced stroke clinician based on the clinical manifestations. The patients with severe head injury, multiple sclerosis, severe cerebral infarction, cerebral hemorrhage, rheumatic diseases or brain malignancy were excluded.

**Fig. 1:**
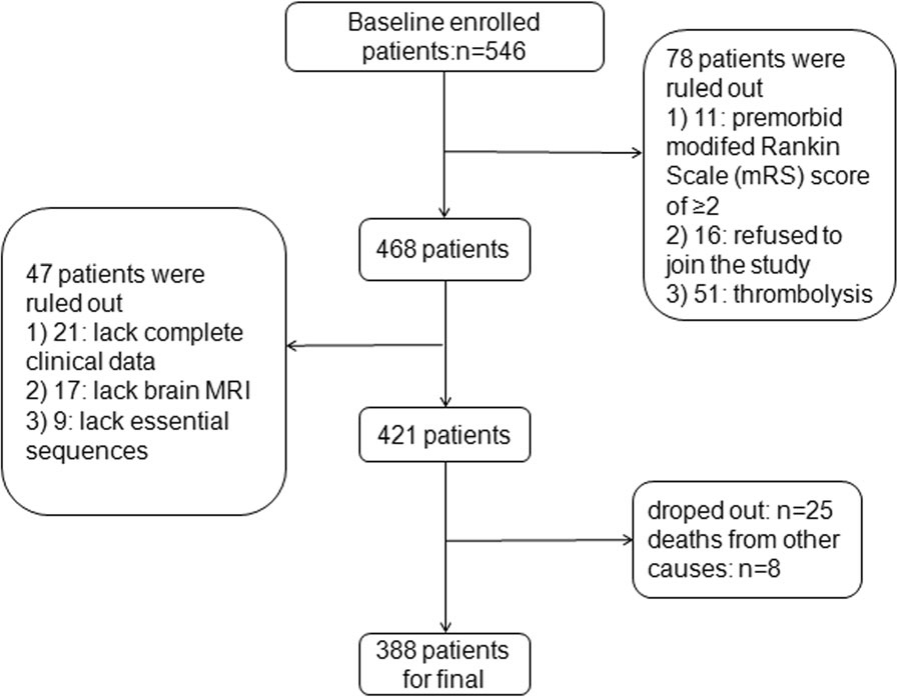
Diagrammatic sketch of the screening process

Finally, a total of 388 consecutive eligible patients were enrolled according to the minor cerebrovascular events definition (National Institutes of Health Stroke Scale Score ≤ 3) and exclusion criteria [16]. All the patients were first-ever minor ischemic stroke. The exclusion criteria were as followed:(1) Premorbid modified Rankin Scale (mRS) score of ≥2 (*n* = 11); (2) Thrombolysis therapy for patients with strokes within 4.5 h of symptom onset (*n* = 51); (3) No baseline MRI (*n* = 17) or one or more of the sequences essential for the calculation of SVD score were missing (*n* = 9); (3) Lacking complete clinical data (*n* = 21); (4) Patients without follow-up data at 3 months missing (*n* = 25); (5) Refused to join in the study (*n* = 16); (6) Deaths from other causes (*n* = 8), with a total of 388 patients left (Fig. 1).

The present study was approved by the ethics committee of Jiangning Hospital.

Affiliated to Nanjing Medical University. Written informed consent was obtained.

from all participants.

### Risk factors

Patients underwent a neurological examination after admission. For determination of the subtype of ischemic stroke, the original TOAST (Trial of ORG 10172 in Acute Stroke Treatment) criteria were used. These 5 major categories of the TOAST classification are as follows: large-artery atherosclerosis (LAA), including large-artery thrombosis and artery-to-artery embolism; cardioembolism (CE); small-artery occlusion (SAO); stroke of other determined cause (OC); and stroke of undetermined cause (UND).

After admission, we collected data of patients’ demographic characteristics and traditional vascular risk factors [8], including age and sex, history of hypertension, history of diabetes mellitus, history of dyslipidemia, history of atrial fibrillation, history of coronary heart disease; history of thrombolysis, history of embolectomy, cigarette or alcohol use. we also collected laboratory results, including results for fasting blood glucose (FBG) and glycosylated hemoglobin (HbA1c), total cholesterol (TC), triglycerides (TG), low density lipoprotein cholesterol (LDL-C), homocysteine (Hcy), lipoprotein-associated phospholipase A2 (Lp-PLA2), high C-reactive protein (H-CRP), and D-Dime.

### Color Doppler ultrasonography (CDU) of the carotid artery

For each patient, we also measured the intima plaques and intima–media thickness (IMT) using a Philips S3000 ultrasound machine (Philips HD20, Netherlands). We observed the formation of intima plaques and measured the IMT using a 7.5- to 10-MHz probe frequency. Plaque formation was diagnosed when the local IMT was > 1.2 mm or > 1.5 times the surrounding IMT [17]. Stable plaques were hard and flat, while unstable plaques were soft, ulcerative, and mixed. The site selected for IMT quantitative measurement was 1.5 cm distal to the carotid artery bifurcation [17]. Two sonographers participated in the evaluation of CDU results.

### Radiologic data

#### MRI acquisition

MRI scanning was performed within 7 days of stroke onset on a 3.0 T MRI scanner (Ingenia, Philips Medical Systems, the Netherlands) with an 8-channel receiver array head coil. High-resolution T1-weighted axial images covering the whole brain were obtained by a 3D-magnetization prepared rapid gradient-echo sequence: TR = 8.1 ms; FA = 90°; TE = 3.7 ms; FOV =240 × 240 mm; acquisition matrix = 240 × 222; gap = 0 mm, thickness = 1.0 mm; number of slices = 170. T2-weighted; TR = 4000 ms; TE =107 ms; FA = 90°; FOV =230 × 230 mm acquisition matrix = 384 × 384; thickness = 1.5 mm; gap = 0 mm, number of slices = 18. DWI-weighted TR = 2503 ms; TE =98 ms; FA =90°; FOV =230 × 230 mm acquisition matrix =152 × 122; thickness = 1.5 mm; gap = 0 mm, number of slices = 18. Susceptibility weighted imaging (SWI)-weighted TR = 16 ms; TE =23 ms; FA =10°; FOV =220 × 180 mm; acquisition matrix = 220 × 180; thickness = − 0.6 mm; gap = 0 mm, number of slices = 200. Additionally, the T2 fluid-attenuated inversion recovery (FLAIR) axial images were obtained with the following parameters: TR = 10,000 ms; TE = 120 ms; FA =110°; FOV =220× 220 mm; acquisition matrix = 336 × 189; thickness = 1.5 mm; gap = 0 mm, number of slices =18.

#### Fazekas score and SVD burden score

**Fig. 2:**
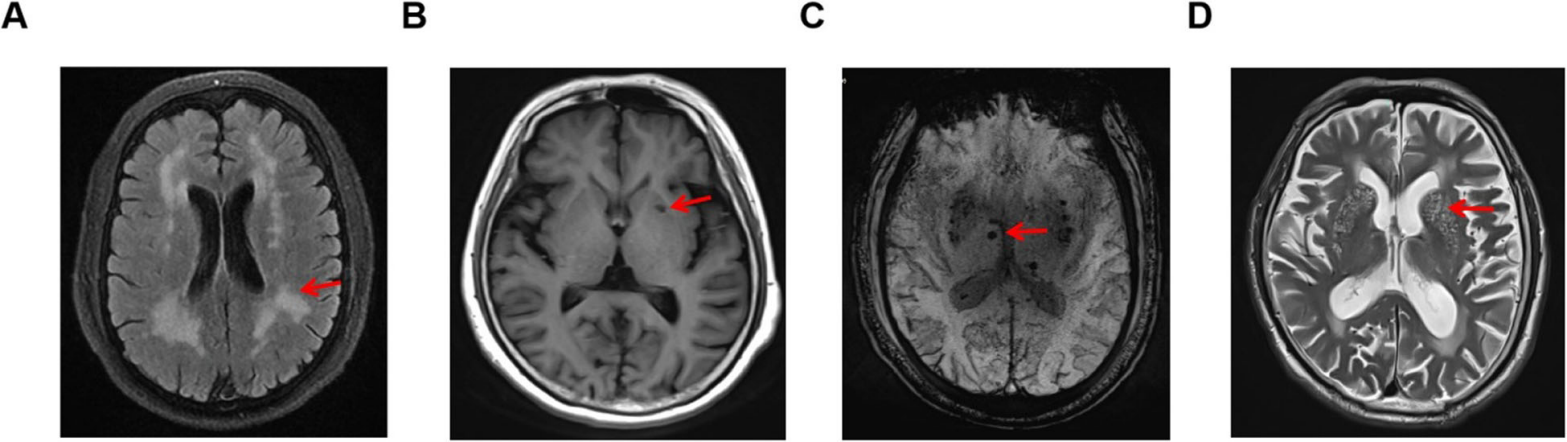
MRI manifestations of cerebral small vessel disease. **a** WMH, high signal lesions on fluid-attenuated inversion recovery sequence; **b** Lacunes, small cerebrospinal fluid-containing cavities larger than 3 mm and smaller than 15 mm in diameter on T1-weighted; **c** CMB, small punctuate areas of hypointensity on susceptibility-weighted imaging measuring up to 10 mm in diameter; **d** PVS, the small deep perforating arterioles as they pass through the deep grey and white matter, made visible on T2-weighted by containing increased fluid of similar signal to CSF

Results are reported in accordance with STRIVE [12]. Fazekas score was used to score WMH [18]. We used the previously described total SVD burden score [13, 14], an ordinal scale (0 to 4) counting the presence of each of the four MRI markers for SVD. In more details, SVD burden score was composed of: (1) WMH was graded using the Fazekas score on FLAIR and divided into periventricular and deep WMH according to the lesion location. If confluent WMH (Fazekas score 2 and 3) were present, one point was awarded (Fig. 2a). (2) lacunar infarcts: presence of one or more lacunes was defined as sharply demarcated hypointense lesions sized between< 15 mm in diameter on T1-weighted images with corresponding hypointense lesions with hyperintense rim on FLAIR [13, 14]. (Fig. 2b). (3) CMB were defined as round hypointense lesions on SWI-weighted gradient echo-images with a diameter < 10 mm. CMB were then divided to lobar versus deep [18]. If ≥1 deep or lobar CMB were present one point was awarded (Fig. 2c). (4) PVS were defined as smooth margin, round, oval, or linear-shaped lesions, ≤3 mm, with signal intensity equal to cerebrospinal fluid (CSF) on T1-weighted images [19, 20]. We counted PVS in the most affected hemisphere. One point was awarded if 30 or more PVS were present at any of the locations (Fig. 2d). Two experienced neurologists and a neuroradiologist participated in the evaluation of MRI results.

### Patient outcomes

We rated the 90-day modified Rankin Scale (mRS) outcome and dichotomized as good functional outcome (mRS 0–1) and poor functional outcome (mRS 2–6) at 90 days [21]. Stroke progression was defined as a worsening deficit during hospitalization compare with NIHSS score at the baseline assessment [22]. A recurrent stroke was defined as a new sudden focal neurological deficit (vascular, > 24 h), which occurs at any time between the initial attack and 90-day follow-up mandating repeat imaging [22]. These outcome events were reviewed in detail by 2 experienced neurologists and a neuroradiologist, and a consensus decision was made on progression or recurrence of stroke [21, 22].

### Statistical analysis

Statistical analysis was conducted using SPSS software (Version 17.0, SPSS Inc., USA). Mean (standard deviation) or median (interquartile range) measurements were used to describe continuous variables which had a normal or non-normal distribution. Frequencies were used to describe categorical variables. Normality was confirmed by the Kolmogorov-Smirnov test. Inter-group comparisons for a normally distributed.

data were performed using the independent sample t test. The χ^2^ tests, Mann-Whitney and the Kruskal–Wallis tests were used to assess categorical variables and non-normally distributed continuous variables. Multiple linear regression analysis was used to explore association of SVD burden and WMH with poor outcome, progression, and recurrence at 90 days controlling for age, gender, vascular risks and admission NIHSS. Furthermore, univariate and multivariate logistic regression analysis was used to ascertain the likelihood that patients will develop poor-outcome after 90 days. Variables with a *P* < 0.05 in univariate regression analyses and other risk factors related to prognosis of minor cerebrovascular events were included in the multivariate logistic-regression models. *P* < 0.05 was considered statistical significance.

## Results

### Baseline characteristics *According to Classification of* mRS score

Totally, 388 patients were included in the final analysis with 317 (81.70%) patients had good outcome and 71(18.30%) patients had poor outcome. Mean age upon admission was 66.54 ± 11.15 years, and 61% were female. In the SVD burden score, 289(74.48%) cases had WMH, 57 (14.69%) cases had CMB, 321 (82.73%) cases had lacunes, and 264 (68.04%) cases had PVS. With significant differences in age, admission NIHSS, LP-PLA2, lacunes, Fazekas score (either periventricular WMH score or deep WMH score) and Fazekas score after 3 months (either periventricular WMH score or deep WMH score) between the good outcome and poor outcome (*P* < 0.05). No significant differences were found in other baseline characteristics (The details were showed in Table 1).

**Table 1:** Baseline characteristics classified according to *(mRS 0-1;2-6)*

Variables	Good outcome (mRS Score 0–1) at 90 d, ***n*** = 317	Poor outcome (mRS Score 2–6) at 90 d, ***n*** = 71	***P*** Value
Age, y, mean ± SD	65.50 ± 11.14	71.23 ± 9.98	< 0.01^∗^
Women, n (%)	198 (62%)	39 (55%)	0.239
Admission NIHSS, median (IQR)	1.25 (1–2.25)	2.25 (2–3)	< 0.01^∗^
Past medical history, n (%)			
Hypertension	262 (83%)	62 (87%)	0.337
Coronary artery disease	38 (12%)	13 (18%)	0.154
Atrial fibrillation	38 (12%)	14 (20%)	0.084
Diabetes mellitus	114 (36%)	28 (39%)	0.583
Hyperlipidemia	127 (40%)	35 (30%)	0.449
Stroke	49 (15%)	12 (17%)	0.621
Hematencephalon	32 (10%)	8 (11%)	0.712
Smoking, n (%)	61 (19%)	12 (17%)	0.648
Alcohol, n (%)	48 (15%)	8 (11%)	0.401
Carotid atherosclerosis, n (%)	221 (76%)	38 (78%)	0.838
TOAST classification, n (%)			0.349
Large artery atherosclerosis	69 (55%)	19 (70%)	
Small vessel disease	44 (35%)	8 (30%)	
Cardioembolic	10 (8%)	0 (0%)	
Undetermined	3 (2%)	0 (0%)	
Clinical variables, median (IQR)			
FBG, mmol/L	5.42 (4.86–6.75)	5.63 (4.88–6.65)	0.493
HbAIc, %	6.00 (5.50–7.30)	5.90 (5.60–9.00)	0.203
TC, mmol/L	4.16 (3.59–4.67)	5.42 (2.94–4.81)	0.255
TG, mmol/L	1.31 (0.96–1.86)	1.29 (0.93–1.51)	0.456
LDL-C, mmol/L	2.38 (1.99–2.87)	2.25 (1.45–2.96)	0.070
H-CRP, mg/L	1.70 (0.80–6.93)	3.10 (0.70–18.30)	0.220
LP-PLA2, ng/mL	143.00 (97.00–261.50)	284.00 (208.00–391.00)	< 0.01^∗^
Hcy, umol/L	12.50 (9.90–15.50)	11.30 (10.10–14.60)	0.328
D-Dimer, mg/L	0.34 (0.25–0.53)	0.27 (0.38–0.69)	0.414
SVD burden score, median (IQR)	3 (2–3)	3 (1–3)	0.894
SVD score is 0, n (%)	4 (1%)	2 (3%)	
SVD score is 1, n (%)	60 (19%)	16 (23%)	
SVD score is 2, n (%)	94 (30%)	15 (21%)	
SVD score is 3, n (%)	132 (42%)	30 (42%)	
SVD score is 4, n (%)	27 (9%)	8 (11%)	
WMH, n (%)	227 (18%)	62 (87%)	0.006^∗^
lacunes, n (%)	226 (71%)	59 (83%)	0.042^∗^
PVS, n (%)	219 (69%)	45 (63%)	0.351
CMB, n (%)	46 (15%)	11 (16%)	0.833
Fazekas score, median (IQR)	4 (2.5–6)	5 (4–6)	< 0.01^∗^
Periventricular WMH score	2 (1–3)	3 (2–3)	< 0.01^∗^
Deep WMH score	3 (1–3)	3 (2–3)	0.020^∗^
SVD burden score after 3 months, median (IQR)	3 (2–3)	3 (2–3)	0.667
SVD score is 0, n (%)	4 (1%)	2 (3%)	
SVD score is 1, n (%)	57 (18%)	13 (18%)	
SVD score is 2, n (%)	96 (30%)	18 (25%)	
SVD score is 3, n (%)	133 (42%)	29 (41%)	
SVD score is 4, n (%)	27 (9%)	9 (13%)	
Fazekas score after 3 months, median (IQR)	4 (2.5–6)	5 (4–6)	< 0.01^∗^
Periventricular WMH score	2 (1–3)	3 (2–3)	< 0.01^∗^
Deep WMH score	3 (1–3)	3 (2–3)	0.022^∗^

Note: ^∗^signifificant difference (*P* < 0.05)

*FBG* Fasting blood glucose, *HbAIc* Glycosylated hemoglobin, *TC* Total cholesterol, *TG* Triglyceride, *LDL-C* Low density lipoprotein cholesterol, *H-CRP* High C-reactive protein, *Hcy* Homocysteine, *LP-PLA2* Lipoprotein-associated phospholipase A2, *SVD* Cerebral small vascular diseases, *WMH* White matter hyperintensity, *PVS* Enlarged perivascular spaces, *CMB* Cerebral microbleeds

### Univariate and multivariate logistic regression analysis of poor functional outcome

After 90 days of the minor cerebrovascular events, we observed 71(18.30%) patients had poor outcome. In univariable logistic regression analysis, age (OR, 1.054; 95% confidence interval, 1.026–1.082; *P* < 0.01), LP-PLA2 (OR, 1.005; 95% confidence interval, 1.003–1.008; *P* < 0.01), Fazekas score (OR, 1.501; 95% confidence interval, 1.241–1.816; *P* < 0.01), lacunes (OR, 1.501; 95% confidence interval, 1.016–3.856; *P* = 0.045) and NIHSS upon admission (OR, 1.980; 95% confidence interval, 1.223–1.464; *P* < 0.01) were associated with poor outcome at 90 days. In the multivariable logistic regression analysis, after adjusting for other confounding variables, age (OR, 1.053; 95% confidence interval, 1.009–1.099; *P* = 0.019), LP-PLA2 (OR, 1.006; 95% confidence interval, 1.003–1.009; *P* < 0.01), Fazekas score (OR, 1.343; 95% confidence interval, 1.020–1.770; *P* = 0.036), and NIHSS upon admission (OR, 1.322; 95% confidence interval, 1.163–1.502; *P* < 0.01) were associated with poor outcome at 90 days (Table 2).

**Table 2:** Univariate and multivariate logistic regression analysis of poor outcome at 90 Days

Variables	Univariate		Multivariate	
	OR (95% CI)	***P*** value	OR (95% CI)^a^	***P*** value
Age	1.054(1.026–1.082)	< 0.01^∗^	1.053 (1.009–1.099)	0.019^∗^
Admission NIHSS	1.338 (1.223–1.464)	< 0.01^∗^	1.322 (1.163–1.502)	< 0.01^∗^
LP-PLA2	1.005 (1.003–1.008)	< 0.01^∗^	1.006 (1.003–1.009)	< 0.01^∗^
Fazekas score	1.501 (1.241–1.816)	< 0.01^∗^	1.343 (1.020–1.770)	0.036^∗^
lacunes	1.980 (1.016–3.856)	0.045^∗^	2.317 (0.818–6.561)	0.133

Note: ^∗^signifificant difference (*P* < 0.05)

*LP-PLA2* Lipoprotein-associated phospholipase A2

^a^Odds ratios were adjusted for baseline characteristics of patients

### Association of SVD burden and WMH with poor functional outcome, progression, and recurrence at 90 days

Univariate logistic regression analysis showed higher Fazekas scale score at baseline was associated with poor functional outcome at 90 days (defined as mRS score 2–6) (OR, 1.501; 95% confidence interval,1.241–1.816; *P* < 0.01), but they were not associated with stroke progression, stroke recurrence (*P* > 0.05). Similarly, higher SVD burden score also was not associated with poor outcome at 90 days, stroke progression and stroke recurrence (*P* > 0.05) (Table 3).

**Table 3:** Association of SVD burden score and WMH With poor outcome, Progression, and Recurrence at 90 Days

Variables	OR (95%CI)	***P*** Value
Poor outcome (mRS:2–6):71/388 = 17.64%		
SVD burden score	0.993 (0.757–1.303)	0.961
Fazekas score	1.501 (1.241–1.816)	< 0.01^∗^
Periventricular WMH score	1.825 (1.365–2.441)	< 0.01^∗^
Deep WMH score	1.477 (1.095–1.992)	0.011^∗^
Recurrent stroke:11/388 = 2.84%		
SVD burden score	0.809 (0.434–1.505)	0.503
Fazekas score	1.012 (0.715–1.432)	0.946
Periventricular WMH score	0.673 (0.384–1.179)	0.166
Deep WMH score	2.055 (0.810–5.214)	0.130
Progression stroke:5/388 = 1.29%		
SVD burden score	0.532 (0.211–1.346)	0.183
Fazekas score	1.441 (0.740–2.806)	0.283
Periventricular WMH score	4.102 (0.713–23.581)	0.114
Deep WMH score	0.969 (0.410–2.294)	0.944

Note: ^∗^signifificant difference (*P* < 0.05)

*SVD* Cerebral small vascular diseases, *WMH* White matter hyperintensity

## Discussion

In the present study, we recruited first-ever minor cerebrovascular events patients and follow-up of 90 days. We found there were 71(18.30%) patients had poor outcome after 90 days. In the MRI makers of SVD, just WMH score at baseline was associated with poor functional outcome at 90 days. In the prediction model, we found WMH score and admission NIHSS can as the independent predictor of poor functional outcome at 3 months after stroke onset.

Increasing evidences have showed that the WMH burden is a negative prognostic marker after AIS [6, 10, 23]. Recently, a large study on more than 5000 first-ever ischemic stroke patients showed that higher supratentorial-WMH volumes were associated with higher 3-month mRS scores [24]. Additionally, brainstem-WMH is proved to be an independent predictor of poor outcome after AIS/TIA and this relationship persist after adjustment for important prognostic factors [24].

Importantly, in our analyses, WMH remained independently associated with less functional recovery after controlling for age and other confounding factors, which suggested it is important to understand the pathological mechanism of the influence of WMH on the prognosis of ischemic stroke, but it remains poorly understood so far [25]. It has been hypothesized that WMH could weaken brain plasticity and capacity to compensate for ischemic injury, partly due to a disruption of white matter fiber tract organization and neuronal network integrity [26]. A possible explanation for this could be that patients with severe WMH have hemodynamic impairment of the distal arteries/terminal arterioles and that this produces a tendency for poor ischemic tissue outcomes. Our finding indicates that the burden of WMH may represent a certain degree of brain fragility, and its addition to chronological age determines an individual’s capacity of post-stroke recovery more accurately than chronological age itself. Our study, therefore, could add to the growing body of literatures suggesting that the extent of WMH affects recovery in patients with stroke. Efficient post-stroke recovery entails reorganization of brain networks, which might be compromised in brains with advanced WMH. In addition, the severity of WMH is associated with post-stroke cognitive decline and depression, and these secondary phenomena are likely to synergistically contribute to unfavorable recovery [27].

In recent years, relationship between SVD burden and clinical outcome of stroke patients has drawn attracted much attention. Some studies have shown that the SVD burden is superior to that of any single SVD marker, although no relationship was found in our study. The lack of association between other SVD markers and poor post-stroke outcome may imply that these SVD markers are not detected on conventional imaging or because of a small sample. With the development of imaging technology, other potential SVD markers may be found one by one, such as cerebral microinfarction, which could more comprehensively evaluate the prognosis of stroke patients [16, 28, 29].

In addition, the poor outcome group had a significantly higher NIHSS scores than the good outcome group. NIHSS is a 15-item neurological function scale used in patients with minor cerebrovascular events. The WMH score increased with the severity of neurological impairment and high scores indicate a worse prognosis for minor cerebrovascular events patients.

The design and patient population of this study offer several strengths including the prospective, single center, first-ever stroke cohort, the identical 3 T MRI protocol and the comprehensive outcome assessment used for all patients. In addition, MRI features of SVD were rated according to the standard neuroimaging characteristics of SVD.

Several limitations should be considered. Firstly, the prospective observational study has a possible selection bias, given that patients with severe AIS, contraindications for MRI, and fatal short-term outcome were missed. At the same time, we only chose patients aged 45 to 85 years. Secondly, the relatively small sample size could contribute to the lack of statistical power to detect smaller influences from other clinical or radiographic variables. Thirdly, SVD features were analyzed as classified but not continuous variables and the burden of WMH is just evaluated by Fazekas scores on FLAIR. Quantifying WMH burden may be more accurately predict the prognosis of minor cerebrovascular events and should be applied in the future work. Furthermore, the association of neuroimaging markers of CSVD with long-term outcomes in patients with minor cerebrovascular events has not been confirmed and it requires further specific longitudinal studies on large populations. Anyway, we are continuing to recruit new minor cerebrovascular events patients and follow-up them in different time points to validate our findings.

## Conclusion

In summary, it was found that the WMH was correlated with short-term functional outcome of minor cerebrovascular events patients and can as a neuroimaging maker of predicting poor functional outcome after minor cerebrovascular events, which highlights the crucial influence of white matter structural integrity on stroke outcome. Taking early detection and treatment of WMH into consideration may be beneficial for improving the functional outcome of patients after minor cerebrovascular events.

## Data Availability

Study data are available from the corresponding author upon request.

## Abbreviations

AIS: Acute ischemic stroke
CSF: Cerebrospinal fluid
CE: Cardioembolism
CRP: C-reactive protein
CMB: Lobar and deep cerebral microbleeds
DWI: Diffusion weighted Imaging
FBG: Glucose fasting blood glucose
FLAIR: Fluid-attenuated inversion recovery
HbA1c: Glycosylated hemoglobin
Hcy: Homocysteine
IMT: Intima–media thickness
LAA: Large-artery atherosclerosis
LDL: Low-density lipoprotein
Lp-PLA2: Phopholipase A2
MRI: Magnetic resonance imaging
mRS: Modified Rankin Scale
NIHSS: National institutes of health stroke scale
OC: Stroke of other determined cause
PVS: Enlarged perivascular spaces
SAO: Small-artery occlusion
SVD: Small vascular diseases
SWI: Susceptibility weighted imaging
TIA: Transient ischemic attack
TC: Total cholesterol
TG: Triglycerides
UND: Stroke of undetermined cause
WMH: White matter hyperintensity
